# The Prevalence and Correlates of Gambling in Australian Secondary School Students

**DOI:** 10.1007/s10899-021-10098-z

**Published:** 2022-01-21

**Authors:** Megan Freund, Natasha Noble, David Hill, Victoria White, Tiffany Evans, Christopher Oldmeadow, Nicola Guerin, Robert Sanson-Fisher

**Affiliations:** 1 Health Behaviour Research Collaborative, School of Medicine and Public Health, Faculty of Health and Medicine, The University of Newcastle, Callaghan, NSW 2308 Australia; 2 Priority Research Centre for Health Behaviour, Faculty of Health and Medicine, The University of Newcastle, Callaghan, NSW 2308 Australia; 3 Hunter Medical Research Institute, New Lambton Heights, NSW 2305 Australia; 4 School of Medicine and Public Health, The University of Newcastle, Callaghan, NSW 2308 Australia; 5 Centre for Behavioural Research in Cancer, Cancer Council Victoria, Melbourne, VIC 3004 Australia; 6 School of Psychological Sciences and School of Population and Global Health, University of Melbourne, Parkville, VIC 3010 Australia; 7 School of Psychology, Deakin University, Geelong, VIC 3220 Australia; 8 Clinical Research Design, Information and Statistical Support, Hunter Medical Research Institute, New Lambton Heights, NSW 2305 Australia; 9 Centre for Clinical Epidemiology and Biostatistics, School of Medicine and Public Health, Faculty of Health and Medicine, The University of Newcastle, Callaghan, NSW 2308 Australia

**Keywords:** Gambling, Adolescents, Australia, Problem gambling

## Abstract

Youth gambling is associated with a range of harms. This study aimed to examine, among Australian adolescents, the prevalence of gambling (ever, in the last month, at-risk and problem), the most frequent gambling types and modalities, and to explore the student characteristics associated with gambling in the last month and with at-risk or problem gambling. Students aged 12–17 years from Victoria and Queensland answered gambling questions as part of the Australian Secondary School Alcohol and Drug (ASSAD) Survey in 2017. The ASSAD also included a series of questions about smoking, alcohol and other drug use, and mental health. A total of 6377 students from 93 schools were included in analysis. The prevalence of ever gambling and gambling in the last month was 31% and 6% respectively. Of students who had gambled in the last month, 34% were classified as at-risk and 15% were classified as problem gamblers. The most frequent types of gambling in the last month were horse or dog race and sports betting. Students who gambled in the last month did so most frequently via a parent or guardian purchasing or playing for them, at home or at a friends’ house, and online or using an app. Regression analysis indicated that male gender, having money available to spend on self, alcohol consumption in the last seven days, the number of types of advertisements seen in the last month, and the number of peer or family members who gambled in the last month, were significantly associated with the likelihood of students gambling in the last month. Male gender, some age categories, and exposure to more types of gambling advertising were also significant predictors of being classified as an at-risk or problem gambler. This large study of youth gambling provides data on gambling behaviours and related variables from a large sample of Australian secondary school students. Student characteristics, including male gender and exposure to more types of gambling advertising, were associated with an increased likelihood of gambling in the last month and of being classified as an at-risk or problem gambler. Further implications of the study findings are discussed.

## Background

While most adolescents are unlikely to reach the typical ‘rock-bottom’ associated with adult problem gambling (Dickson et al., 2002), harms associated with gambling are not restricted to adult populations (Delfabbro, 2012; Delfabbro & Thrupp, 2003; Hardoon & Derevensky, 2002; Productivity Commission, 2010; Splevins et al., 2010; Volberg et al., 2010). Studies have consistently indicated that adolescent gambling can lead to harms including: missing or dropping out of school; undermined friendships; family disruptions; and criminal behaviour (Derevensky & Gupta, 2004; Fisher, 1999; Gupta & Derevensky, 1998; Huang et al., 2007; Yeoman & Griffiths, 1996). Adolescent gambling has also been associated with depression, and tobacco, alcohol and other drug use (Delfabbro & Thrupp, 2003; Monaghan & Derevensky, 2008; Scholes-Balog et al., 2016). Adolescents who gamble, particularly those adolescents who are considered to be problem or severe problem gamblers (Productivity Commission, 2010), are at-risk of financial harm as a result of their gambling (Delfabbro & Thrupp, 2003; Monaghan & Derevensky, 2008; Scholes-Balog et al., 2016). There is also some evidence that gambling in early adolescence may be associated with problems with gambling in adulthood (Burge et al., 2004; Griffiths, 2011; Shaffer & Hall, 2001).

Many adolescents have gambled at some point during their lifetime (Barnes et al., 2009; Huang & Boyer, 2007; Shaffer et al., 1999). Meta-analyses of studies conducted in the US and Canada indicated that a median of 85% and 73% of adolescents report lifetime and past-year gambling respectively (Jackson et al., 2008); while a recent international review of 13 Australian studies and 26 international studies (from countries including Canada, the US and UK, Italy, and Finland), reported between approximately 40 and 70% of young people had gambled in the last year (King et al., 2020). Internationally, across countries including the US, Brazil, Hong Kong, Australia, New Zealand, and several European countries, the prevalence of problem gambling among youth is estimated to range from 0.2% to 12% (Calado et al., 2017). Variation in the prevalence estimates of adolescent lifetime, annual, and problem gambling likely reflects differences in the instruments used to assess gambling and problem gambling, the age and gender of respondents, as well as inter-country variations such as gambling legislation, types of gambling available, and access to gambling venues (Calado et al., 2017; Delfabbro et al., 2016). Although access and availability varies between countries, in most North American, European, and Australasian studies, the most prevalent forms of adolescent gambling were lotteries (e.g. scratchies), and betting on card games, games of skill, and sporting events (Delfabbro et al., 2016).

Cross-sectional studies suggest a number of individual, interpersonal and community factors are correlated with increased youth gambling engagement, including relatively low family connectedness (Gupta & Derevensky, 1998; Hardoon et al., 2004) and higher reported gambling among immediate family members (Delfabbro & Thrupp, 2003; Gupta & Derevensky, 1998; Hardoon et al., 2004; Shead et al., 2010). Peer gambling is also a significant factor (Delfabbro & Thrupp, 2003; Purdie et al., 2011; Shead et al., 2010). A 2017 systematic review examined protective and risk factors longitudinally associated with the subsequent development of gambling problems (defined as any measure of problem gambling, pathological gambling or gambling disorder) in young people up to the age of 25 years (Dowling et al., 2017). Early risk factors which showed a small effect size for subsequent problem gambling included: alcohol, tobacco, cannabis, and other illicit drug use; anti-social behaviours (including deviancy and theft); peer antisocial behaviours (including deviancy); depressive symptoms; sensation seeking; and violence. Early risk factors with a small-medium or medium effect size for problem gambling included impulsivity; number of gambling activities; uncontrolled temperament; poor academic performance; and male gender. Problem gambling severity had a strong mean effect size for subsequent problem gambling (Dowling et al., 2017). Early protective factors against problem gambling were: higher parent supervision; fewer social problems; and higher socio-economic status (Dowling et al., 2017). Other protective factors which have been identified for adolescent problem gambling include higher resilience and greater family cohesion (Derevensky & Gilbeau, 2015).

Gambling is a common activity in Australia, with around 64% of adults gambling at least once a year (Armstrong and Carroll, 2017). For those under the age of 18 years, gambling in a commercial premise or using an online gambling site is illegal (Delfabbro & Thrupp, 2003; HealthWest Partnership, 2010). However, adolescents can gamble informally at home or among friends. There is also some evidence that adolescents in Australia participate in activities that are not legally accessible to minors, facilitated by parents or older siblings (Delfabbro et al., 2005). Emerging environmental issues that are likely to impact on adolescent gambling prevalence are the expansion of gambling promotion and opportunities to gamble (Delfabbro et al., 2016), including online gambling. The rise of smart phone and other internet-based technologies has provided unprecedented access to gambling activities (Hing et al., 2014a, b). In 2015, 80% of Australian adolescents aged 14 to 17 had a smart phone, 65% had used a mobile phone to go online (Victorian Responsible Gambling Foundation, 2017), 97% accessed at least one social media platform (Gainsbury et al., 2015a, b), and 60% of young people who had gambled had done so online (Victorian Responsible Gambling Foundation, 2017). This is despite age restrictions intended to minimise access to gambling products to those under the age of 18. Exposure to advertising is also indicated to increase the likelihood of engaging in gambling activities (Clemens et al., 2017; Derevensky et al., 2010). Although there are restrictions on gambling advertising during children’s television programs and sporting events in Australia (Australian Communications and Media Authority, 2019a), adolescents and children are not shielded from this promotion. For example, children may be exposed to gambling advertising during sport outside of restricted times (e.g. after 8.30 pm) (Australian Communications and Media Authority, 2019b) and many young people report exposure to gambling promotions online through social media platforms such as Facebook, Twitter and YouTube (Gainsbury et al., 2015a, b).

Given the emergence of these environmental issues, we undertook this study to explore the prevalence of gambling and problem gambling among a population based sample of Australian adolescents. Specifically, this study examined, among Australian secondary school students aged 12–17 years, the:

- Prevalence of gambling (ever, in the last month) by age and gender;
- Prevalence of problem gambling for those who gambled in the last month by age and gender;
- Prevalence of gambling types and modalities for those who gambled in the last month and;
- Associations between gambling in the last month and problem gambling with student characteristics.

## Methods

### Study Design

Gambling questions were included in the cross-sectional, triennial Australian Secondary School Alcohol and Drug (ASSAD) Survey for the states of Victoria and Queensland in 2017. The ASSAD survey is a national survey of 20,000 to 30,000 secondary students aged between 12 and 17 years old (Guerin & White, 2018). The study design and survey were approved by the relevant State and institutional Human Research Ethics Committees (HRECs), including the University of Newcastle HREC (Ref: H-2017–0102).

### Sample and Procedure

Sampling was two-staged. Firstly, a random sample of schools, stratified by education sector (Government, Catholic, and Independent sectors) to reflect state-wide distributions was developed for each participating state. Secondly, within schools, classes of students in Years 7 to 12 were randomly selected from the school student roll to complete the ASSAD survey. Classes selected by ability or performance were excluded in order to maintain the representativeness of the sample. Data collection took place during the 2017 academic school year. A passive (opt-out) parental consent process was used for Government and independent schools, while active parental consent was required in Catholic schools. On the day agreed with the school, researchers attended the school to administer the pencil-and-paper questionnaire to selected students. Further details regarding the ASSAD methodology have been published elsewhere (Guerin & White, 2018). Only Victorian and Queensland students completed the ASSAD with the additional gambling items, and comprise the sample for this current study.

### Measures

The gambling items were developed through an iterative process stemming from an extensive literature review, advice from experts in adolescent youth gambling and smoking research, and pilot testing of the selected items with a group of adolescents. Prior to answering the gambling-related questions, students were given the following definition of gambling: ‘Gambling is when you pay in your own money knowing that you could lose all of it or, possibly, win back even more than you paid in. There are lots of ways to gamble, for example on the results of races, sports, card games, lotteries, raffles, on machines like “pokies”, tipping competitions and sweepstakes.’

#### Ever Gambled and Gambled in the Last 30 days

Students were asked ‘Have you ever bet any money on any form of gambling?’ (yes/no). Students who answered in the affirmative, were asked if they had gambled in the past 30 days (yes/no).

#### Types of Gambling Activities

Students who had ever gambled answered questions regarding gambling activity types. Students indicated, for each gambling activity, whether they had ever gambled on that activity (e.g. ever gambled playing card games), and whether they had gambled on that activity in the last month.

#### Modality of Gambling

Students who had ever gambled answered questions regarding how they gambled (e.g. online, at a pub or club, or facilitated by another person). The modality items were based on work by King et al. and Gainsbury et al. that explored social gaming and gambling (Gainsbury et al., 2015a, b; King et al., 2014). The items examining other peoples’ facilitation of student gambling were based on measures of adolescent smoking (White et al., 2005) and alcohol use (Hearst et al., 2007).

#### Problem Gambling

Students who had ever gambled were screened for problem gambling using the 12 item Diagnostic Statistical Manual IV (Multiple Response format) adapted for Juveniles (DSM-IV-[MR]-J). This tool is frequently used by youth gambling researchers (Stinchfield, 2011), and has demonstrated reasonable levels of reliability and validity (Fisher, 2000; O'Neil et al., 2003; Rossen, 2001). In the current study, response options were revised to a dichotomous scale (yes/no). This is consistent with other Australian research (Delfabbro & Thrupp, 2003; Delfabbro et al., 2005), and previous work suggesting the ‘yes/no’ response scale is more easily answered than frequency response options for this age range (Purdie et al., 2011). Respondents who had gambled in the last 30 days were classified as follows: (a) non-problem gamblers (did not endorse any of the diagnostic criteria); (b) at-risk gamblers (responded ‘yes’ to between one and three of the diagnostic criteria); and (c) problem gamblers (responded ‘yes’ to four or more of the diagnostic criteria). The classification of ‘problem gambler’ is consistent with the scoring system used by Fisher (1999), where a respondent with four or more ‘yes’ responses is classified as a problem gambler (Fisher, 1999).

#### Gambling in the Social Environment

All students were asked to complete questions regarding peer and family gambling based on items developed by Delfabbro and Thrupp (Delfabbro & Thrupp, 2003). Participants were asked to select which people they knew who had gambled in the last 30 days (mother/caregiver 1, father/caregiver 2, brother or sister, other relative, one of your best friends, someone else you know).

#### Exposure to Gambling Promotion

Exposure to advertising was measured through an adaptation of Hing et al.’s exposure to sports advertising scale (Hing et al., 2014a, b). The scale was modified to include non-sports promotions, including promotions on social media. Students were asked to indicate their awareness of a range of advertisements or promotions for gambling in the past 30 days (e.g. as ads for gambling on TV, radio, billboards, and live studio crosses to gambling operators).

#### Student Characteristics

Students self-reported their: postcode; age; gender; main language spoken at home; money to spend on self per week; perceived school achievement; and attendance at school on previous school day. Students answered questions on their use of tobacco and alcohol in their lifetime, past year and past month (yes/no). Students indicated the number of alcoholic drinks or cigarettes they had consumed on each of the previous seven days. A student who drank alcohol on at least one day in the past seven was defined as a user of alcohol. A smoker was defined as a student who had smoked on more than two days in the last seven days. Students indicated if they had ever used other drugs (including cannabis, hallucinogens, ecstasy, heroin, cocaine, amphetamines, or steroids). Responses were combined to indicate any illicit substance use or none. Students also indicated if they had ever been diagnosed or told by a doctor or nurse that they have a mental health condition. Student’s home postcode was used to classify students’ residential location according to the Accessibility and Remoteness Index of Australia (ARIA+), as either major city or other (inner regional, outer regional, remote, very remote) (Australian Bureau of Statistics, 2018). Level of socioeconomic disadvantage was also based on student postcode using the Socio-Economic Indexes for Areas (SEIFA) Index of Relative Socio-economic Disadvantage (IRSD) decile classifications (Australian Bureau of Statistics, 2017).

### Analysis

Students presumed to be disengaged, i.e., with a large amount of missing data or whose responses were wildly exaggerated, were removed from the data set prior to analysis. Categorical variables were summarised as frequencies, and percentages of non-missing responses. Continuous variables are presented with mean, standard deviation, median, minimum and maximum values. Gambling prevalence (ever and within the last 30 days) and problem gambling classifications for students who had gambled in the last month, were compared across age and gender. Due to the large sample size, the Rao-Scott F-adjusted chi-square statistic was used for statistical comparisons by student age and gender. The design effect included strata state (Queensland and Victoria), school type (Independent, Government and Catholic) and clustering for individual schools. Multiple testing was accounted for by controlling the false discovery rate at 5%. All *p*-values less than 0.023 were considered statistically significant. All statistical analyses were programmed using SAS v9.4 (SAS Institute, Cary, North Carolina, USA).

#### Factors Associated with Student Gambling Behaviour and Problem Gambling

Examination of factors associated with student gambling behaviours in the last 30 days, and with students being classified as at-risk or problem gamblers, was undertaken using a multivariate approach. Two separate multivariate logistic regressions were performed with gambling in the last 30 days, and classification as an at-risk or problem gambler, as the dependent variables. Independent variables for both regressions were: gender (male vs female); age (12–13, 14, 15, 16, and 17 years; 12 and 13 year olds were combined into one category due to small numbers of at-risk or problem gamblers among these age groups); socioeconomic disadvantage (SES deciles); rurality (major city vs other); main language spoken at home (English vs other); money to spend on self per week ($20 increments); perceived school achievement (above average, average, below average); smoking status (smoker vs non-smoker); alcohol consumption in past 7 days (yes/no); any illicit drug use in lifetime (yes/no), any mental health conditions (yes/no); number of people they know gamble (0, 1, 2, 3, 4+); number of gambling advertising/promotion types seen in the last 30 days; and visited a venue where people were gambling in the last 30 days (yes/no).

## Results

A total of 93 schools participated in the ASSAD survey in 2017 (57 in Victoria and 36 in Queensland). The most common reasons for non-participation of secondary schools were multiple survey requests from various organisations (unable to meet all requests), timing of request (close to exams, school camps, students participating in work experience) and lack of staff time to coordinate survey. Sixty-three (68%) of the participating schools were Government schools, 14 (15%) were Catholic schools and 16 (17%) were Independent schools. This was broadly representative of the distribution of schools across the three education sectors within each state.

### Student Characteristics

In total, over 7000 students took part in the survey. Students who were missing responses to all of seven core gambling module questions were removed from the dataset (n = 707), leaving a total of 6489 students. A further 112 students did not answer the first gambling question (have you ever gambled?) and were also removed from the dataset, resulting in a final sample size of 6377 students for analysis. Students who responded ‘yes’ to the first gambling question (have you ever gambled?) but were missing a response to the gambling in the last month question were assumed not to have gambled in the last month (n = 272).

The demographics of participants are shown in Table 1. Just over half of the sample were female (56%), and the majority of students were located in major cities (65%) and spoke English at home (81%). The largest proportion of the sample were aged 16 years (23%), and 13 years (19%). On average students saw four different types of gambling advertisements in the last month (e.g. ads on television, online, billboards etc.). Most students reported that they did not know anyone who had gambled in the last month (62%), while more than one in ten students reported that they knew at least two people in their peer group and/or family who had (14%). Over one-third of the sample had visited a venue where gambling was available in the last month (39%).

**Table 1:** Student characteristics, N = 6377

Characteristic	N	%
*Gender*		
Female	3600	56%
*Age*		
12	485	8%
13	1193	19%
14	1027	16%
15	1083	17%
16	1475	23%
17	1114	17%
*Socioeconomic disadvantage: SEIFA IRSD*		
Deciles 1–2 (most disadvantage)	866	14%
Deciles 3–4	1343	21%
Deciles 5–6	1190	19%
Deciles 7–8	1537	24%
Deciles 9–10 (least disadvantage)	1390	22%
*Geographic area*		
Major city	4141	65%
Inner regional	1365	22%
Outer regional	749	12%
Remote/very remote	78	1%
English main language spoken at home	5125	81%
*Money to spend on self per week*		
None	892	14%
$10 or less	1141	18%
$11–$20	1139	18%
$21–$60	1390	21%
$61–$100	698	11%
Over $100	1049	17%
*Perceived school achievement*		
A lot above/above average	2499	39%
Average	3278	52%
A lot below/below average	550	9%
Smoked on ≥ 3 days of past 7 (yes)	219	3%
Drank alcohol in past 7 days (yes)	1201	19%
Any illicit drugs used in lifetime (yes)	1284	20%
Any mental health condition (yes)	804	13%
*Number of people know who gamble*		
None	3665	62%
One	1413	24%
Two	471	8%
Three	241	4%
Four or more	136	2%
*Number of different types of advertisements seen*		
Mean (SD)	4 (3)	
Median (min, max)	3 (0, 11)	
Visited venue where gambling occurs in last month (yes)	2292	39%

Columns may not add to total due to missing data

### Gambling Prevalence

A total of 31% of students (n = 1942) reported that they had ever gambled, and 408 (6%) reported they had gambled in the previous month (see Table 2). The prevalence of ever gambling differed by age category (*p* < 0.001), and was more common in male students (37%) than female students (25%; *p* < 0.001). Similarly, the prevalence of gambling in the past 30 days differed by age category (p = 0.022) and more male students had gambled in the last month (9%) than females (5%; *p* < 0.001). The prevalence of ever and past month gambling was also calculated while excluding “softer” forms of gambling (tipping competitions, sweeps, bingo, lottery tickets, scratch cards, and raffle tickets; data not shown). The prevalence of engaging in hard gambling ever was 23%, and in the last month was 4%. Similar differences in hard gambling prevalence were seen across age and gender, although the prevalence of hard gambling in the last month was no longer significantly different across age categories (*p* = 0.051). Twenty-eight percent of males versus 19% of females had ever gambled on a hard gambling activity, and 6% of males versus 3% of females had gambled on a hard gambling activity in the last month.

**Table 2:** Prevalence of gambling by age and gender, N = 6377

	Age in years							
	12 N = 485	13 N = 1193	14 N = 1027	15 N = 1083	16 N = 1475	17 N = 1114	Total N = 6377	*p*^#^
*Ever gambled*								
Male (N = 2777)	51 (24%)	162 (30%)	164 (35%)	213 (41%)	251 (42%)	189 (43%)	1030 (37%)	< 0.001^*^
Female (N = 3600)	47 (17%)	123 (19%)	162 (29%)	145 (26%)	255 (29%)	180 (27%)	912 (25%)	< 0.001^*^
Total	98 (20%)	285 (24%)	326 (32%)	358 (33%)	506 (34%)	369 (33%)	1942 (31%)	< 0.001^*^
*Gambled in last month*								
Male (N = 2777)	5 (2%)	33 (6%)	42 (9%)	49 (10%)	60 (10%)	50 (11%)	239 (9%)	0.003^*^
Female (N = 3600)	5 (2%)	24 (4%)	30 (5%)	34 (6%)	44 (5%)	32 (5%)	169 (5%)	0.404
Total	10 (2%)	57 (5%)	72 (7%)	83 (8%)	104 (7%)	82 (7%)	408 (6%)	0.022^*^

^*^Indicates statistically significant differences (using *p* ≤ 0.023)

^#^p values refer to age group comparisons

### Problem Gambling

Across the whole sample, there were 296 students who had ever gambled but did not answer enough questions to be classified using the DSM-IV-JR. Of the students who could be classified (n = 6081), 73% (n = 4435) were classified as non-gamblers, 18% (n = 1068) as non-problem gamblers, 8% (n = 459) as at-risk gamblers, and 2% (n = 119) were classified as problem gamblers.

Table 3 shows the problem gambling classifications for those students who reported gambled in the last 30 days (n = 408). Among students who had gambled in the last 30 days and could be classified (n = 391), 51% were non-problem gamblers, 34% were at-risk gamblers, and 15% were classified as problem gamblers. The difference in the prevalence of problem gambling across age groups was non-significant. However, the prevalence of problem gambling in those who had gambled in the last month was significantly higher for males (22%) than females (6%; *p* < 0.001).

**Table 3:** Problem gambling classification for students who reported gambling in the last 30 days by age and gender (N = 391)

Classification:	Non-problem gambler	At-risk gambler	Problem gambler	*p*
*Age*				0.074
12–13yrs (N = 64)	37 (58%)	20 (31%)	7 (11%)	
14yrs (N = 67)	44 (66%)	17 (25%)	6 (9%)	
15yrs (N = 77)	29 (38%)	32 (42%)	16 (21%)	
16yrs (N = 103)	56 (54%)	29 (28%)	18 (18%)	
17yrs (N = 80)	34 (43%)	33 (41%)	13 (16%)	
*Gender*				< 0.001^*^
Males (N = 228)	91 (40%)	86 (38%)	51 (22%)	
Females (N = 163)	109 (67%)	45 (28%)	9 (6%)	
*Total (N* = *391)*	200 (51%)	131 (34%)	60 (15%)	

There were 17 students who could not be classified due to missing data on the DSM-IV-JR. Percentages are presented as row proportions for those who reported gambling in the last 30 days. Classification of problem gambling was based on Fisher (2000) and ACER 2011 scoring of the DSM-IV-[MR]-J with modified yes/no response options. ^*^Indicates statistically significant differences (using *p* ≤ 0.023)

### Type of Gambling

For students who had ever gambled (n = 1942), the most frequent types of gambling activities were buying raffle tickets (53%), horse or dog races (48%), and instant scratchies (44%). The most frequent types of gambling activities for students who had gambled in the last 30 days (n = 408) are shown in Table 4. The most frequent gambling activity was horse or dog races (38%) followed by betting on popular sports (football, cricket etc., 29%), then personal skill games (26%). Over 20% of students who had ever gambled, and 7% of students who gambled in the last month, reported having gambled on poker machines and on casino-type games.

**Table 4:** Type and modality of gambling for students who gampled in the last 30 days (N = 408)

	n	%
*Type of gambling*		
Horse or dog races	154	38%
Sports games (e.g. football, rugby, cricket)	118	29%
Personal skill games (e.g. pool, darts, video games)	105	26%
Bought raffle tickets	101	25%
Card games (e.g. poker, blackjack, 21)	81	20%
Instant scratchie card	80	20%
Lottery ticket (e.g. Keno, Tattslotto, Powerball)	78	19%
Tipping competitions	68	17%
Sweeps	58	14%
Bingo for prizes or money	45	11%
Two up	28	7%
Poker machines (pokies)	29	7%
Casino games (e.g. roulette, craps or dice)	29	7%
Other	32	8%
*Modality of gambling*		
Parent/guardian purchased or played for me	213	52%
At home or the home of a friend	200	49%
Online via a website (e.g. on a laptop or computer)	116	28%
Using an app on a tablet or mobile	95	23%
Another relative purchased or played for me	81	20%
At the racecourse	75	18%
At a TAB	62	15%
At a pub or club	59	15%
At a newsagent	57	14%
A friend purchased or played for me	53	13%
Brother/sister purchased or played for me	39	10%
Over the phone	40	10%
Someone else purchased/played for me	34	8%
At a casino	21	5%

### Gambling Modality

Among students who had ever gambled, the most frequent gambling modalities were gambling at home or at a friend’s home (41%), a parent or guardian purchasing or playing for the student (40%), and online via a website (15%). The different modalities of gambling for students who reported gambled in the last month are shown in Table 4. The three most frequently reported modalities for gambling in the last month were the same as for ever gambling: a parent or guardian purchasing or playing for the student (52%), followed by gambling at home or at a friend’s home (49%), and online gambling (28%). Almost a quarter of students who had gambled in the last month reported gambling using an app on a tablet or mobile phone (23%). A substantial proportion of students who had gambled in the last month did so at a racecourse (18%), TAB (15%), or at a pub or club (15%).

### Factors Associated with Student Gambling in the Past Month and Problem Gambling

#### Gambling in the Past Month

Table 5 presents the results of the multivariable regressions. After controlling for the other variables in the model, gender, money available to spend on self, alcohol consumption in the last seven days, the number of types of gambling advertisements seen in the last month, and the number of peer or family members who gambled in the last month, were significantly associated with student’s gambling in the last month. The odds of gambling in the last month were 51% less for females (OR 0.49; 95% CI: 0.39, 0.62). A $20 increase in the amount of money a student had to spend on themselves was associated with 6% increase in the odds of gambling (OR 1.06; 95% CI: 1.01, 1.10). Drinking alcohol in the last seven days was associated with a 52% increase in the odds of gambling in the last month (OR 1.52; 95% CI: 1.16, 2.00). Being exposed to one additional type of gambling advertisement was associated with a 6% increase in the odds of gambling (OR 1.06; 95% CI: 1.02, 1.09). A greater number of people (parents, siblings, friends etc.) gambling in the last month in a student’s social environment was associated with increasing odds of gambling (ORs ranged from 5.00 to 36.13).

**Table 5:** Multivariate association between key student factors and gambling in the last 30 days and at-risk or problem gambling

	Gambled in last month (N = 5574)				At-risk or problem gambling** (N = 357)			
Factor	OR	Lower 95% CI	Upper 95% CI	*p-*value	OR	Lower 95% CI	Upper 95% CI	*p-*value
*Sex*				< 0.001^*^				< 0.001^*^
Male	Reference					Reference		
Female	0.49	0.39	0.62		0.33	0.19	0.56	
*Age*				0.94				0.021^*^
12–13	Reference				Reference			
14	1.10	0.75	1.60		0.58	0.23	1.43	
15	1.20	0.74	1.95		1.84	0.75	4.52	
16	1.05	0.60	1.85		0.90	0.35	2.32	
17	1.00	0.56	1.79		1.34	0.54	3.32	
SES deciles (linear)	1.02	0.96	1.07	0.52	1.00	0.89	1.13	0.95
*Geographic area*				0.92				0.99
Major city	0.98	0.67	1.44		0.99	0.47	2.09	
Other	Reference				Reference			
*English mainly spoken at home*				0.97				0.03
Yes	1.01	0.69	1.46		0.43	0.21	0.91	
No	Reference				Reference			
Money to spend on self per week (linear increase in $20 increments)	1.06	1.01	1.10	0.012^*^	1.05	0.96	1.14	0.30
*Self-considered school achievement*				0.51				0.18
A lot above average/above average	1.36	0.78	2.36		0.53	0.25	1.12	
Average	1.26	0.72	2.21		0.53	0.25	1.11	
Below average/a lot below average	Reference				Reference			
*Smoked on ≥ 3 days of past 7*				0.84				0.46
No	Reference				Reference			
Yes	1.06	0.63	1.77		1.66	0.44	6.30	
*Drank alcohol in past 7 days*				0.002^*^				0.35
No	Reference				Reference			
Yes	1.52	1.16	2.00		1.33	0.73	2.42	
*Illicit drugs used in lifetime*				0.61				0.50
No	Reference				Reference			
Yes	1.09	0.78	1.52		0.81	0.44	1.49	
*Any mental health condition*				0.27				0.36
No	Reference				Reference			
Yes	1.19	0.87	1.62		1.38	0.69	2.76	
*No. advertisement types (linear 0–11)*	1.06	1.02	1.09	< 0.001^*^	1.10	1.03	1.18	0.008^*^
*No. people know who gamble (linear)*				< 0.001^*^				0.11
None	Reference				Reference			
One	5.00	3.58	6.97		1.74	0.89	3.41	
Two	7.58	5.22	11.01		1.60	0.72	3.53	
Three	13.00	8.59	19.68		2.28	1.01	5.12	
Four or more	36.13	21.22	61.50		2.50	1.25	4.98	
*Visited venue where gambling occurs*				0.87				0.30
Yes	1.02	0.80	1.31		0.78	0.49	1.24	
No	Reference				Reference			

^*^Indicates statistically significant differences (using *p* ≤ 0.023); ^**^Uses all available cases for those who gambled in the last month

#### Problem Gambling

After controlling for the other variables in the model, gender, age, and the number of types of gambling advertisements seen in the last month, were significant predictors of being classified as an at-risk or problem gambler. Females were 67% less likely to be classified as an at-risk or problem gambler than males (OR 0.33; 95% CI: 0.19, 0.56). Age was a significant predictor in the multivariable model, however, the odds of problem gambling in the older age categories did not differ significantly from the 12–13 year old age group (ORs include 1). Being exposed to one additional type of gambling advertisement was associated with a 10% increase in the odds of being classified as a problem or at-risk gambler (OR 1.10; 95% CI: 1.03, 1.18).

## Discussion

Across this large sample of Australian secondary school students drawn from two Australian states, approximately one-third (31%) had ever gambled, and 6% reported gambling in the last month. The prevalence of ever gambling in this study was relatively low compared to some other studies of Australian adolescents, where estimates range from 41% up to 80% gambling in the last year (Delfabbro et al., 2005; Jackson et al., 2008; Splevins et al., 2010), and up to 90% gambling in their lifetime (Moore & Ohtsuka, 2001). Rates are also lower than those typically reported in international studies, with a recent review indicating between 40 and 70% of young people from countries including Australia, the US and UK, Canada, Italy, and Finland engaged in gambling activities in the last year (King et al., 2020). In the current study, when only “hard” gambling activities were considered, prevalence rates reduced to 23% of adolescents ever gambling, and 4% of adolescents gambling in the last month, on hard activities. Previous Australian and international studies have not distinguished between “hard” and “soft” forms of gambling, with King et al., 2020 noting that adolescents may have difficulty determining whether certain activities (such as raffles) are considered to be gambling, and many studies including raffles, scratch cards, and lottery tickets within the definition of gambling. One argument for including softer forms of gambling in youth prevalence studies is the notion that gambling is a slippery slope, which may start with soft gambling and naturally proceed to hard forms of gambling (Kamis et al., 2010). In line with previously reported patterns of youth gambling (Delfabbro et al., 2016), gambling was more common for male students than female students, and the prevalence of gambling tended to increase with age. This study represents the largest and arguably most representative quantitative study of gambling prevalence ever conducted among Australian adolescents, and is the first to include data from multiple States. As noted by King et al., many of the previous Australian studies are not nationally representative (King et al., 2020). As such, the current study provides potentially the most reliable and up to date estimates of gambling prevalence among Australian adolescents.

Despite the relatively low prevalence of gambling in adolescents in our study, 10% of all students were classified as being at-risk (8%) or as problem gamblers (2%). This rate is comparable with that reported in other Australian studies, where the prevalence of problem gambling ranged from < 1% (Delfabbro & King, 2011) up to 4% (Delfabbro et al., 2005) and 5%(Purdie et al., 2011). It is also within the wide range of problem gambling reported internationally (Calado et al., 2017). We also found that among those students who had gambled in the last month, almost half were classified as either at-risk or problem gamblers (15%). Previous studies which also examined the prevalence of problem gambling among adolescents who reported recent gambling (i.e. in the last month) were not able to be identified for comparison.

In line with previous research (Kristiansen & Jensen, 2014), betting on sports games and personal skill games were among the most frequently reported types of gambling. In contrast to previous reports, the most common type of gambling among students who had gambled in the last month was betting on horse or dog races; while participating in lotteries or buying scratchie was less frequent compared to previous findings (Kristiansen & Jensen, 2014). The popularity of horse and dog race gambling aligns with findings reported by Moore & Ohtsuka for a sample of students from Melbourne (Victoria). They note this likely reflects the popularity of the Melbourne Cup horse race held annually in Victoria, marked by a state-wide public holiday, where family sweeps and betting by parents for children are common (Moore & Ohtsuka, 2001).

The most common modes of gambling among our sample included for a parent or guardian to purchase or play for the student, and gambling at home or a friend’s house. In addition, a significant proportion of those who gambled in the last month did so at a racecourse, a betting shop, or pub/club, despite gambling in such venues being legally restricted to those over the age of 18 years. These findings highlight the potentially important role parents and family play in facilitating gambling activities for children. Indeed, as noted above, Moore and Ohtsuka point out that the Melbourne Cup is traditionally seen as a ‘family betting day’ (Moore & Ohtsuka, 2001). A number of other Australian studies noted that adolescents who gambled on lottery products and TAB or other racing activities typically did so with adult assistance (Delfabbro et al., 2005; Lambos et al., 2007). However the current study also found that nearly 30% of students who had gambled in the last month did this via an online platform, and almost a quarter had gambled using an app on a tablet or mobile phone. This contrasts with earlier Australian findings (conducted prior to 2011) where internet gambling was the least popular form of gambling among adolescents (Delfabbro et al., 2005; Jackson et al., 2008; Splevins et al., 2010). It is likely that our results reflect the growing accessibility and opportunities for adolescents to gamble presented by new technology such as websites and gambling apps (King et al., 2020; Messerlian et al., 2004).

Similar to previous studies we found that being male, having more money available to spend on self, having consumed alcohol in the last week, seeing a greater number of types of advertisements in the last month, and knowing a greater number of peer or family members who had gambled in the last month, increased the likelihood of student’s gambling in the last month. Being male and seeing a greater number of types of gambling advertisements in the last month, was also associated with being an at-risk or problem gambler. These results echo previous findings which indicate that boys are more likely than girls to gamble, and to display problem gambling behaviours (Blinn-Pike et al., 2010; Delfabbro et al., 2016). In contrast to previous work (Calado et al., 2017; Delfabbro et al., 2016), age was not significantly associated with the likelihood of gambling, and the prevalence of problem gambling did not increase uniformly with age. A number of Australian studies have reported similar inconsistencies in problem gambling across age (Splevins et al., 2010), or non-significant differences in problem gambling prevalence by age or grade-level (Delfabbro et al., 2005).

Previous work has also indicated that adolescent problem gambling is generally associated with other risk-taking behaviours such as smoking, alcohol and substance use, as well as poor mental health (Blinn-Pike et al., 2010; Delfabbro et al., 2016). In our sample, only alcohol consumption in the last week was associated with increased odds of gambling in the last month, while the other risk-taking behaviours including recent smoking and lifetime illicit drug use were not. Contrary to expectations, none of the other risk-taking behaviors (i.e. smoking, lifetime use of illicit substances) or having a mental health condition were associated with the being classified as an at-risk or problem gambler.

Results of this study highlight the particular vulnerability of males to gambling activities and problem gambling, and reinforce the important role played by family and peers, as well as exposure to gambling promotions, in influencing adolescent gambling behavior. Previous work has confirmed high recall of gambling advertising among young people, especially for television and social media advertising (Thomas et al., 2018), and that higher exposure to gambling advertising is related to multiple gambling outcomes, including youth gambling frequency and problem gambling (Clemens et al., 2017; Derevensky et al., 2010; Gavriel Fried et al., 2010). Similar to alcohol and tobacco use (Jackson & Bartholow, 2020; Pierce et al., 2012; Sargent & Babor, 2020; Weitzman & Lee, 2020), advertisements appear to play a social role in the process of developing gambling behaviour among adolescents (Gavriel Fried et al., 2010). Such findings reinforce the need to regulate and restrict gambling promotion to adolescents (Hing et al., 2014a, b; Parrado-González & León-Jariego, 2020). The findings are also consistent with previous work indicating that the frequency of parental gambling is associated with adolescent gambling behavior (McComb & Sabiston, 2010). Parents often approve of and are involved in their children’s gambling activities, and may unknowingly contribute to adolescent gambling behaviours including problem gambling (Felsher et al., 2003). It appears that many Australian parents purchase or play gambling activities for their secondary school aged children. This suggests that prevention programmes for adolescents need to be accompanied by public education and awareness for parents and adults about the types of gambling-related problems experienced by adolescents (Felsher et al., 2003). Recent Australian research points to the need to challenge adult perceptions of lottery products (e.g. lottery tickets and scratchies) being ‘harmless’, and the importance of discouraging parents from giving such products to their children as gifts (Booth et al., 2020; Wilkinson, 2020).

### Strengths and Limitations of the Study

This is the largest and most recent representative study of youth gambling that has been conducted in Australia, and provides reliable baseline data on gambling prevalence and associated variables for over 6,000 students across two States. The embedding of the gambling questions in the triennial ASSAD will allow for future monitoring of changes in gambling prevalence and problem gambling among Australian adolescents over time. Such monitoring is valuable in order to track changes, identify likely causes and implications, and adjust policy and program priorities, as has been done in Australia for tobacco and alcohol use since 1987 (White et al., 2008, 2017). However, the study has a number of limitations. Firstly, as recommended by the study’s Expert Advisory Panel, activities that may be considered ‘softer’ forms of gambling, including raffles, tipping competitions, and sweeps, were included in the definition of gambling given to students. The definition and gambling activities included were consistent with several other recent Australian (Abbott et al., 2016; Hing et al., 2016) and international studies (Horch & Hodgins, 2013). Secondly, all variables collected in the questionnaire were self-reported, and as such are subject to potential recall error and social desirability biases.

## Conclusion

This large study of youth gambling in provides recent baseline data on gambling behaviours and related variables for a large representative sample of Australian secondary school students. Up to date data are particularly important given the evolving landscape of gambling, with new forms of gambling and technologies such as smartphones and similar devices making gambling widely accessible, highly visible, and socially accepted (King et al., 2020; Riley et al., 2021). In addition to the accessibility of gambling online or using an app, the current results particularly highlight the vulnerability of young males to gambling and problem gambling, and the important role of advertising and peer and family influences on gambling behaviours. Our findings suggest the need to further reduce youth exposure to advertising as well as to educate peers or family about the impact of their gambling behaviour on young people.

## Declarations

### Availability of data and material

The datasets used during the current study are available from the corresponding author on reasonable request.
